# Mental health research and evaluation in multicultural Australia: developing a culture of inclusion

**DOI:** 10.1186/1752-4458-7-23

**Published:** 2013-10-07

**Authors:** Harry Minas, Ritsuko Kakuma, Lay San Too, Hamza Vayani, Sharon Orapeleng, Rita Prasad-Ildes, Greg Turner, Nicholas Procter, Daryl Oehm

**Affiliations:** 1 Centre for International Mental Health, Melbourne School of Population and Global Health, The University of Melbourne, Melbourne, Australia; 2 Victorian Transcultural Mental Health, St Vincent’s Hospital Melbourne, Melbourne, Australia; 3 Mental Health in Multicultural Australia, Brisbane, Australia; 4 Queensland Transcultural Mental Health Centre, Metro South Hospital and Health Service, Brisbane, Australia; 5 University of South Australia, Adelaide, Australia

## Abstract

**Introduction:**

Cultural and linguistic diversity is a core feature of the Australian population and a valued element of national identity. The proportion of the population that will be overseas-born is projected to be 32% by 2050. While a very active process of mental health system reform has been occurring for more than two decades - at national and state and territory levels - the challenges presented by cultural and linguistic diversity have not been effectively met. A key area in which this is particularly an issue is in the collection, analysis and reporting of mental health data that reflect the reality of population diversity. The purpose of this study was to examine: what is known about the mental health of immigrant and refugee communities in Australia; whether Australian mental health research pays adequate attention to the fact of cultural and linguistic diversity in the Australian population; and whether national mental health data collections support evidence-informed mental health policy and practice and mental health reform in multicultural Australia.

**Methods:**

The study consisted of three components – a brief review of what is known about mental health in, and mental health service use by, immigrant and refugee communities; an examination of national data collections to determine the extent to which relevant cultural variables are included in the collections; and an examination of Australian research to determine the extent to which immigrant and refugee communities are included as participants in such research.

**Results:**

The review of Australian research on mental health of immigrant and refugee communities and their patterns of mental health service use generated findings that are highly variable. The work is fragmented and usually small-scale. There are multiple studies of some immigrant and refugee communities and there are no studies of others. Although there is a broadly consistent pattern of lower rates of utilisation of specialist public mental health services by immigrants and refugees the absence of adequate population epidemiological data prevents judgments about whether the observed patterns constitute under-utilisation. There are virtually no data on quality of service outcomes. The examination of national data collections revealed multiple gaps in these data collections. The review of papers published in four key Australian journals to determine whether immigrants and refugees are included in mental health research studies revealed a high rate (9.1%) of specific exclusion from studies (usually due to low English fluency) and a much higher rate of general neglect of the issue of population diversity in study design and reporting.

**Conclusions:**

While there are many positive statements of policy intent in relation to immigrant and refugee communities in national mental health policies and strategies there is virtually no reporting by Commonwealth or State and Territory governments of whether policies that are relevant to immigrant and refugee communities are effectively implemented. It is not possible, on the basis of the data collected, to determine whether immigrant and refugee communities are benefiting from the mental health system reforms that are being actively carried out. The majority of Australian mental health research does not adequately include immigrant and refugee samples. On the basis of the findings of this study eight strategies have been recommended that will contribute to the development of a culture of inclusion of all Australians in the national mental health research enterprise.

## Introduction

*All people have certain fundamental human rights. Membership in our society confers on all Australian residents, including people with mental health problems or mental disorders, certain rights, roles and responsibilities. Australia is a diverse society comprising people from a wide variety of cultural and linguistic backgrounds. Every Australian needs to be encouraged to maintain his or her mental health and to work towards the prevention of mental health problems and mental disorders. Some may require assistance to do this. The Commonwealth, State and Territory Governments are now seeking to redress inequities in Australian society by way of social justice strategies*[1]*.*

Cultural and linguistic diversity is a core feature of the Australian population [2] and a valued element of national identity. If net overseas migration continues at the current rate the overseas-born component of the Australian population will increase from the current proportion of more than 25% to around 32% in 2050 [3]. The existing cultural and linguistic diversity of the population, and the arrival of immigrants and refugees from a very wide range of source countries [2] will continue to present challenges for all forms of service delivery, including mental health services, into the foreseeable future.

The process of mental health system reform has been occurring in all States and Territories since the 1950s. The development of a National Mental Health Strategy in 1992 [4], endorsed by the Commonwealth and all State and Territory governments, has given considerable impetus to the reform process. There has been a major shift from hospital to community-based service delivery [5], substantial increases in the mental health workforce, improved access to mental health services in primary care, improved mental health literacy in the general population [6], substantial increases in participation in decision-making by people with mental illness and their families and support persons [7], and a continuing move from a focus on medical treatment to recovery-oriented mental health [8,9] and psychosocial support services.

Key components of the national reform process have been a clear statement of rights and responsibilities [1], the development of national standards for service delivery [9], a commitment to evidence-informed policy development, service delivery and reporting of progress against policy intent [7], and a focus on service outcomes [10]. In all relevant Commonwealth, State and Territory mental health policy documents culturally and linguistically diverse (CALD) populations have been identified as warranting particular attention in order to ensure equity [11].

However, it is not clear whether immigrant and refugee communities - particularly those who do not speak English, the most recently arrived and refugees, who are among the most vulnerable – have benefited from this process of major mental health system reform.

The collection and analysis of mental health data is central to moving toward equity in mental health. Without data on the population distribution of mental health and mental illness, the patterns of service use by different sections of the population, and the quality of outcomes of health service contact, unjust inequalities remain invisible. Mental health and mental health service inequities need to be made visible to enable evidence-informed policy development, mental health service design and delivery, and clinical and recovery practice. Comprehensive and reliable data are essential to evaluate the degree to which policies and programs enhance equity, provide direction for research into root causes, and guide new strategies for promoting health [12].

The purpose of this study is to examine:

•What is known about the mental health of immigrant and refugee communities in Australia;

•Whether Australian mental health research pays adequate attention to the fact of cultural and linguistic diversity in the Australian population and;

•Whether national mental health data collections support evidence-informed mental health policy and practice and mental health reform in multicultural Australia.

The recommendations, based on the main findings, are intended to contribute to the development of a culture of inclusion of all Australians in the national mental health research enterprise.

## Cultural diversity in Australia

*“Whatever the future holds for Australia, history suggests it will be inextricably bound up with immigration.”*[13]

**Figure 1:**
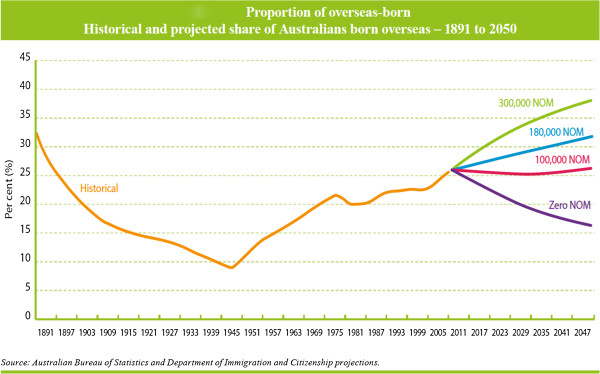
**Proportion of Overseas-born.** Historical and projected share of Australians born overseas - 1891 to 2050.

The cultural and linguistic diversity of the Australian population has been shaped by Australia’s unique history. At the end of World War II, the population was approximately seven million, of whom 10% were overseas-born [14]. Since the end of the Second World War the proportion of overseas-born has steadily increased [3]. (Figure 1) The first post-war wave of migration consisted predominantly of new arrivals from Europe [14]. In each of the subsequent decades, an additional one million immigrants arrived [14]. By June 2011, the population was 22.3 million of whom 26% were born overseas and an additional 20% had at least one overseas-born parent [2]. Over the past ten years, the overseas-born population has increased by 23.1% [15].

In 2011 persons born in the United Kingdom continued to be the largest country-of-birth group (5.3% of the total population), followed by people born in New Zealand (2.5%), China (1.8%), India (1.5%), Vietnam (0.9%) and Italy (0.9%) [2].

Migration source countries have continued to change, with a reduction in migration from the United Kingdom and significant increases in migration from New Zealand, China and India. The most rapid population growth between 2001 and 2011 was for persons born in Nepal (with an average annual increase of 27%), Sudan (17.6%), India (12.7%), Bangladesh (11.9%) and Pakistan (10.2%) [15].

**Figure 2:**
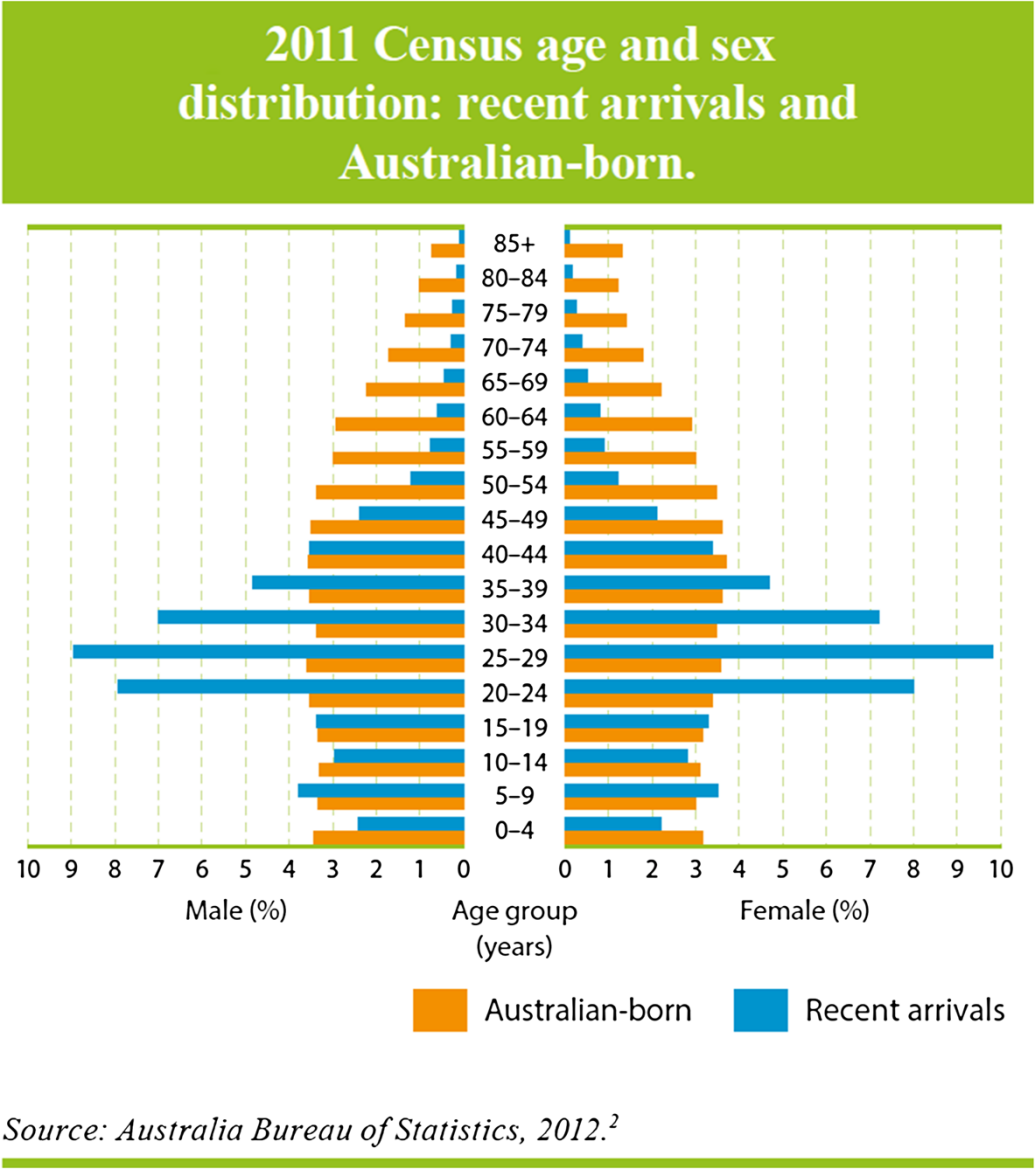
2011 Census age and sex distribution: recent arrivals and Australian-born.

**Figure 3:**
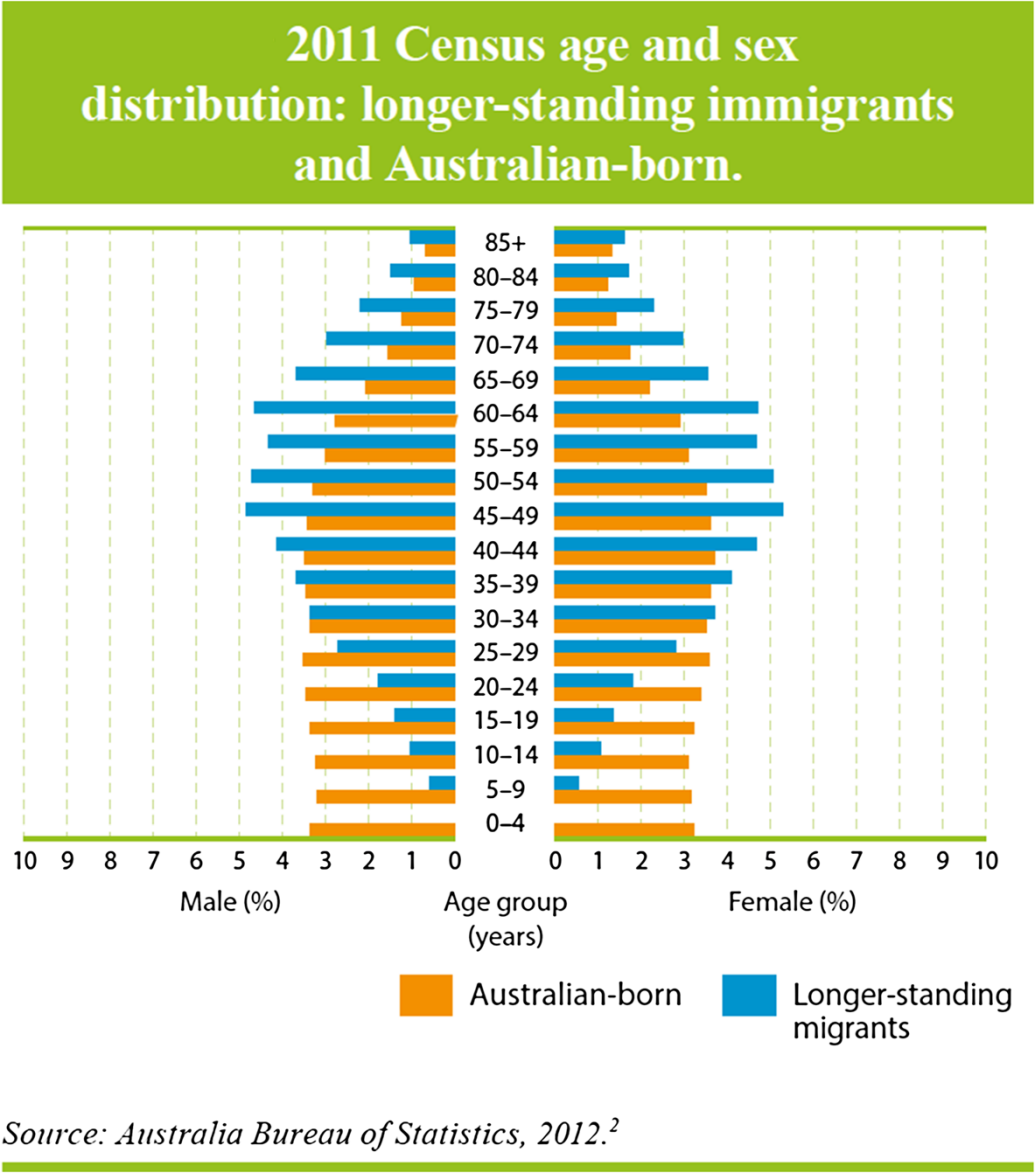
2011 Census age and sex distribution: longer standing immigrants and Australian-born.

Recent immigrants are younger than the general population [2] (Figure 2) while longer-standing immigrants are older than the general Australian population (Figure 3) [2]. The relative youth of recent arrivals is important for mental health. Adolescence and young adulthood is the peak period of onset of most mental disorders. This is also a period for many immigrants when they are dealing with the many stresses associated with migration and settlement. For longer-settled immigrants the key issue is the mental disorders of old age. Disorders associated with cognitive impairment are a substantial challenge for families and for the mental health system, particularly when they include deterioration in the person’s capacity to communicate in English.

The majority of recent immigrants (67%) and almost half (49%) of the longer-standing immigrants speak a language other than English at home [2]. For longer-settled immigrants, Mandarin (4.3%), Cantonese (4.2%), Italian (3.7%) and Vietnamese (3.2%) were the most common languages spoken at home other than English, while for recent immigrants, Mandarin (10.8%), Punjabi (3.7%), Hindi (3.3%) and Arabic (3.0%) were the languages other than English most frequently spoken at home [2].

Approximately half (51%) of the longer-settled immigrants reported that they could speak English very well, while only 2.6% reported that they could not speak English at all. Among recent arrivals (past 10 years) 43% reported that they speak English very well, while 3.1% reported not speaking English at all [2]. There is, as expected, wide variation in level of English fluency among country-of-birth groups.

While cultural and linguistic diversity represents a significant challenge, the development of mental health services that are responsive, accessible, culturally appropriate and effective in meeting the needs of people with mental illness and their families and support persons, is not a distraction from the’ core business’ of mental health services. Working through the process of reforming services so that they are capable of meeting the needs of a culturally diverse society will have the direct benefit of making those services more flexible and responsive to the needs of all members of the Australian community.

## Mental health reform in Australia

*Our community is rich in diversity. It embraces cultural and religious differences. This brings many strengths and opportunities, but we also need to recognise the challenges faced at times by some within our community. There should be demonstrated cultural competency in the planning and delivery of responsive mental health services*[16]*.*

An analysis of Commonwealth, State and Territory mental health policies, and of specific transcultural mental health policies developed in New South Wales, Queensland, Victoria, and Western Australia, revealed that statements such as the above are common [11]. Commonwealth, State and Territory mental health policies were examined for their relevance to mental health system responses to depression in immigrant and refugee communities. Specialised State ‘transcultural mental health’ policies provided comprehensive policy coverage of issues relevant to mental health and immigrant and refugee communities. Key topics were identified from these transcultural mental health policies and used to analyse each of the general Commonwealth, and State and Territory mental health policies. There was a highly variable degree of attention to issues relevant to immigrant and refugee communities. Commonwealth policies contained a relatively comprehensive coverage of issues. Areas that were unrepresented or under-represented included: providing information which supports access; interpreters/language services; coordination of care; support for ethnic community workers; data collection; and service utilisation. More recent policies tended to include a clearer focus on immigrant and refugee communities and highlighted the need for improvement in the evidence base for all forms of mental health activity in relation to immigrant and refugee communities. Policies developed have tended to repeat the same aspirations concerning immigrant and refugee communities, as illustrated by the quote above from the 4th National Mental Health Plan [16].

While such statements of policy intent are a welcome acknowledgement of the need to focus on cultural and linguistic diversity, two key questions remain. To what extent are such statements of policy intent included in policy implementation plans or used to establish funded programs? Do Commonwealth, State and Territory governments and mental health service agencies report progress against such statements of policy intent?

A survey seeking information on whether services were addressing depression in immigrant and refugee communities was sent to 1,480 organisations in capital cities and major regional towns across Australia [11]. The organisations surveyed included mental and general health service providers, Divisions of General Practice, public health units, Local Governments, Migrant Resource Centres, transcultural mental health services, refugee services and ethnic community organisations. Relevant programs were analysed in relation to reported strategies and activities, barriers, supports, perceived role in relation to depression in immigrant communities, partnerships, and program involvement of immigrant communities, people with mental illness and their families and support persons. From the 1,480 organisations to which questionnaires were sent 422 organisations (28%) responded to the survey. A total of 46 programs were identified that specifically addressed mental health in immigrant and refugee communities and a further eight programs reported that they were mainstream mental health programs that had made some adaptation to be more responsive to the needs of immigrant and refugee communities. “Direct clinical services, such as counseling, psychotherapy, psychiatric case management, psychological rehabilitation, day activity programs, self-help and mutual support groups for those with mental disorders, were all reported by mainstream mental health organisations. These were regarded as available to the whole of the community with no particular adaptations of programs to accommodate the varied needs of ethnic minority communities” [11].

The continuing process of mental health system reform in Australia, particularly over the past two decades, has resulted in major changes in the ways in which professionals and communities think about mental health and illness, in the ways in which mental health services are designed and delivered, and in the level of priority accorded to mental health by Commonwealth, State and Territory governments. The general population is more knowledgeable about mental health and illness [6] and more likely to seek mental health treatment and care [5], services are much more community-focused, the importance of primary care in service delivery has been recognised and supported, participation of people with mental illness and their families and support persons in decision-making has been considerably strengthened, and there has been a strong and deliberate move to recovery-oriented service delivery. While these achievements have led to Australia being regarded as a world leader in mental health system reform, it is recognised that there is still much to be done [17].

It is clear that policy-makers are aware of the relevance of cultural and linguistic diversity and of the need to take such diversity into account when framing mental health policy, and when designing mental health services. However, there is very little implementation of those components of mental health policies that relate to the particular needs of immigrant and refugee communities.

Lack of implementation is all but invisible because reports of progress in implementation of Commonwealth, State and Territory mental health policies generally say nothing about immigrant and refugee communities. A recent and important example is the National Mental Health Report 2010 [7]. The report “summarises the progress of mental health reform undertaken over the fifteen years of the National Mental Health Strategy, and provides a view of trends and performance at the national and State and Territory levels over the period spanning the First, Second and Third Mental Health Plans from 1993 to 2008.” This *Summary of 15 Years of reform in Australia’s Mental Health Services under the National Mental Health Strategy 1993–2008,* like the ten National Mental Health Reports that preceded it, has nothing to say about immigrant and refugee communities.

As a result of such neglect, and exclusion from implementation and reporting processes, there may well be persistent disparities in availability of and access to mental health services, quality of care, and mental health outcomes for people from immigrant and refugee backgrounds [18-22]. The lack of comprehensive and reliable data on mental health of immigrant and refugee communities means that disparities and inequities that do exist are all but invisible.

## Mental health of immigrant and refugee communities in Australia

*“As a group, people born overseas have health characteristics that are different from the rest of the population. The mortality and morbidity patterns of migrants can be influenced by both their country of origin and where they currently live, and by the process of migration itself.”*[23]

What is known about mental health of immigrant and refugee communities in Australia and what are the major gaps in our knowledge? To answer this question a search of publications reporting mental health research carried out in Australia between 1963 and August 2012 was carried out to identify studies that included immigrant or refugee communities and that focused on immigrant and refugee mental health issues. The search yielded 214 original research articles reporting findings on mental health issues from samples of participants from immigrant and refugee background. The findings below are from this review.

### Prevalence of mental disorders

Knowledge of prevalence of mental disorders is essential for several reasons. Without reliable estimates of prevalence of different types of mental disorders in CALD communities it is impossible to say anything about the scale of mental health problems in different populations. It difficult to evaluate whether attempts to improve population mental health are effective and a good investment. It is also difficult to determine whether differences in rates of service utilisation by those sub-groups are due to differences in prevalence or due to other factors, such as obstacles to service access. Reliable prevalence estimates are important to inform mental health policy and service design and delivery.

The National Survey of Mental Health and Wellbeing conducted by the Australian Bureau of Statistics [5] provides the best available estimate of the prevalence of mental disorders across Australia. Information from the survey is very important in formulating government mental health policies and decisions about mental health services.

The 2007 National Survey of Mental Health and Wellbeing collected information from 8,841 Australians aged 16–85 years. The survey provides information on the prevalence of selected mental disorders (Anxiety, Affective, and Substance Use disorders), sex and age distributions, comorbidity, and the extent of impairment of core activities and health service utilisation.

Demographic characteristics relevant to people from immigrant and refugee backgrounds included: Country of Birth, Year of Arrival, Country of birth of mother or father, and Proficiency in spoken English. The classification of countries used is the Standard Australian Classification of Countries (SACC).

While the Australian Bureau of Statistics provides an overview of the findings from the survey [5] most of the detailed analyses that are available have been carried out by researchers with access to the survey dataset [2,10,19,21,24-39]. None of these studies has analysed findings relevant to immigrant and refugee participants in the survey. (There was one study examining access to mental health care by people from non-English-speaking background using data from the first, 1997, National Survey of Mental Health and Wellbeing [40]).

The results relevant to immigrant and refugee participants reported from the 2007 national survey are prevalence rates by country of birth (Australia or Overseas) and year of arrival of immigrants. Respondents born outside Australia were found to have lower prevalence of anxiety, affective, substance use disorder, and of any 12-month mental disorder [5] (Table 1). Those most recently arrived (in the decade prior to the survey) have the lowest rates of disorder.

**Table 1:** Prevalence by Country of Birth and Year of Arrival, 2007 National Survey of Mental Health and Wellbeing

	**Country of birth**		**Year of arrival to Australia**		
	**Born in Australia**	**Total born Overseas**	**Arrived before 1986**	**Arrived 1986-1995**	**Arrived 1996-2007**
	**% of sample^**	**% of sample^**	**% of sample^**	**% of sample^**	**% of sample^**
Anxiety disorders	15.4%	11.6%	13.4%	11.3%	8.7%
Affective disorders	6.6%	5.1%	5.4%	4.4%	5%
Substance use disorders	6%	2.8%	*1.6%	*5.7%	*3.0%
Any 12 month mental disorder	21.8%	15.1%	15.8%	17.5%	12.5%
No 12-month mental disorder	78.2%	84.9%	84.2%	82.5%	87.5%

Source: Australian Bureau of Statistics, 2008 [5].

* Estimate has a relative standard error of 25% to 50% and should be used with caution.

^ Sample includes only persons aged 16–85 years.

The ABS reports that only 2.2% of the potential sample could not participate in the 2007 Survey of Mental Health and Wellbeing due to language difficulties, which includes people with language barriers such as deafness or disability. Given the level of English fluency that would be required to respond to the survey, this is difficult to reconcile with 2011 Census of Population and Housing [2] figures that only 51% of longer-settled immigrants and 43% of recent arrivals reported that they could speak English “very well’, while 2.6% and 3.1% reported that they could not speak English at all.

Although there have been varied findings on whether the prevalence of common mental disorders in immigrant and refugee populations are the same, lower or higher than in the Australian-born population [18,31,37-39,41,42] the weight of evidence from studies in other countries and Australian studies suggests that prevalence of mental illness in immigrant communities is similar to that in host populations, and that prevalence across particular country of birth groups is highly variable.

The results of prevalence studies vary widely according to the disorder being studied, particular ethnic or country of birth groups, and the location of the study. It is possible to find reports of higher [42] and lower [37,43-45] prevalence of various disorders in various groups, and numerous studies where no difference has been found between immigrant groups and host populations [18,46-58]. For young immigrants, evidence showed that fewer mental health problems were reported by immigrant adolescents compared to non-immigrant adolescents [59]; nevertheless, children of immigrants were found to be similar with children of Australian-born in terms of their mental health problems [60-62].

The rates of depression, anxiety and post-traumatic stress disorder were between three and four times higher among Tamil asylum seekers in Australia than the rates of these problems among immigrants [63,64], and Ziaian and colleagues found that young refugees had increased risk of depressive symptoms [65]. Substantial proportions of Burmese refugees in Australia experienced mental health problems including depression, anxiety and post-traumatic stress disorder (PTSD) [66], while Vietnamese refugees had lower prevalence of mental disorders than the Australian-born sample. Similar prevalence of PTSD was found in these two groups. PTSD was diagnosed in 50% of Vietnamese refugees and 19% of Australians with any mental disorders [44]. Additionally, refugees and asylum seekers are particularly vulnerable to self-harm and suicidal behaviours. In Australia the prevalence of self-harm among detained asylum seekers was reported to be higher than in the general population and among prisoners [67].

Consistent with studies from other countries, Australian studies have shown that immigrant suicide rates tend to reflect the rates of their country of birth, an association that is particularly evident in males [68]. In general, suicide rates are higher among immigrants born in countries that have higher suicide rates such as Western, Northern, and Eastern European countries, while rates are lower in immigrant groups from countries with lower suicide rates including those in Southern Europe, the Middle East, and South-East Asia [69-71].

### Determinants of mental health problems

A key goal of mental health research is to understand the determinants of mental health and illness – both risk and protective factors – and to develop effective health promotion, illness prevention and early intervention, and effective treatment and psychosocial support service programs [72]. A number of factors have been identified as potentially important risk or protective factors for mental illness among immigrant groups in Australia. The extent to which these factors are important across all immigrant groups is not known because findings are based on a very small number of studies with only very few immigrant groups.

Several factors have been found to be associated with increased risk of mental disorder among immigrants. They include limited English proficiency [73], separated cultural identity [74], loss of close family ties [75], lack of opportunity to make effective use of occupational skills [76], trauma exposure prior to migration, and the many stresses associated with migration and adjustment to a new country [77].

Protective factors include religious belief and observance, younger age at migration, better English proficiency, a higher sense of personal control, stronger social support and higher self-efficacy [78,79]. A survey of 1,139 immigrant and refugee people in two rural and two metropolitan areas in Victoria focused on their experiences of racism and its association with psychological distress [80]. Approximately two-thirds of participants had experienced racism in the previous 12 months and reported that this had adversely affected their mental health. The extent of experiences of racism was positively correlated with level of psychological distress.

Risk of suicidal behaviour among immigrants is influenced by factors including living circumstances in the host country [69,71], experiences in the country of origin [71] and low socio-economic status [70]. Strong family ties, religious adherence and maintenance of traditional values may lead to lower suicide rates in immigrants [81].

The mental health of refugees and asylum seekers is negatively affected by pre-migration trauma [82,83], long-term detention [82,84-86], temporary protection [82,87,88], restriction of access to services [89], human rights violations [89,90], exposure to threats of different kinds [90] and fear for family remaining in the country of origin [91].

A sense of belonging to family and community and perceived social support are positively associated with better mental health among refugees [92,93]. Exposure to violence and threats to their parents are important risk factors for child refugees, whereas stable settlement and social support have a positive effect on psychological functioning [94,95].

Pre-migration trauma and longer periods of detention [82,96,97] are associated with increased risk of suicidal behaviours among refugees. The experience of detention increased the likelihood of mental health problems such as anxiety, depression, and PTSD, as well as self-harm behaviours and suicidal ideation [96].

Although there are more studies of refugees and asylum seekers than of most other immigrant sub-groups in Australia samples are generally very small and not all studies use rigorous methods. This is particularly true for studies of people who are in or have been in immigration detention. Conclusions drawn from such studies need to be treated with caution.

### Explanatory/conceptual models of mental health and illness

A number of studies have explored explanatory models of mental health and illness in individual immigrant and refugee groups in Australia [98-106]. The objectives and design of these studies have been variable. Although the findings of these studies are of considerable theoretical value there has been no systematic attempt to explore the practical significance of the findings – to inform clinical practice, community engagement, and use of health services, mental health service design or mental health policy. A comprehensive program of research that examines the relationship between explanatory models of mental health and illness, conceptions of appropriate mental health service response, and service design and delivery issues is needed to inform the development of culturally appropriate and effective mental health services.

### Mental health service utilisation

*This statement recognises that people with mental health problems or mental disorders should have access to services and opportunities available in Australian society for people of a similar age with equity and justice. Access to, and availability of, appropriate services requires consideration of specific needs and ideally is not limited by cultural and ethnic barriers, or by communication capacities and skills including language*[1].

In an analysis of the 1997 National Survey of Mental Health and Wellbeing [40] people from English-speaking and Non-English Speaking Backgrounds (NESB) were equally likely to experience anxiety disorders and affective disorders, but the latter were less likely to experience substance-use disorders and any mental disorder. People from non-English speaking and English-speaking backgrounds were equally likely to use services for mental health problems and there was no difference between birthplace groups in terms of their likelihood of reporting that their needs were fully met (perceived need for care). Country of birth data for immigrants were aggregated to the level of born in an English-speaking country and born in a non-English-speaking country so that no conclusions could be drawn about specific country of birth groups.

In studies of particular country of birth groups the likelihood of receiving treatment for mental disorder is influenced by immigrants’ country of birth [18,107]. For example, migrants from Greece diagnosed with mental disorder were more likely to receive treatment than Australians; however, the opposite was found in immigrants from UK or Ireland or South East Asia [18]. Nevertheless, in general, immigrants are under-represented in the populations who utilise mental health service in Australia [108-110]. The key barriers identified are stigma and shame attached with mental illnesses [111-113]. Other hindrances including limited access to mental healthcare, the quality of care received, limited knowledge of services, communication difficulties, confidentiality concerns, lack of trust in service providers, service constraints and discrimination [112,114].

Refugees and asylum seekers in Australia have low hospital admission rates for treatment of mental disorder and low access to mental health care services [65,115]. This was due to the presence of a range of impediments including Medicare ineligibility, unaffordable health care costs and the impacts of social, financial and psychological difficulties [116,117]. Shame or fear of being judged by others and treatment provider, and fear of hospitalisation have been reported as barriers to access to health care services among refugees [111].

These barriers were found to be greater in refugees from higher education background and longer residency in Australia [111]. Young refugees have been reported to be more likely to seek helps from friends than from professional sources [118]. The reasons for not turning to professional help included low concern about mental health, poor knowledge of mental health and service, distrust of services, stigma associated with mental health problems and other social and cultural factors [118].

In Victoria, relative to the Australian-born, immigrant and refugee communities have consistently been found to have lower rates of access to public community and inpatient mental health services [19,20], a higher proportion of involuntary admissions, and higher proportions who are diagnosed with a psychosis [19-21]. Similar findings have been reported from New South Wales [57], Queensland and Western Australia [119].

The low rates of access by immigrant and refugee communities may be due to lower prevalence of mental illness in immigrant and refugee communities than in the Australian-born population. This is not consistent, however, with research showing that overall community prevalence of mental illness in immigrant samples is similar to that of Australian-born samples [18,42], or that levels of mental illness may be higher in refugee communities [120] than in host communities.

A pattern of under-utilisation of mental health services by particular groups may point to systematic inadequacies in service systems, raise important questions concerning the need for service reform, community attitudes towards and beliefs about mental illness and psychiatric treatment, barriers to service access, difficulties in diagnosis, and racism.

### Mental health outcomes

*The consumer has the right to have services subjected to quality assurance to identify inadequacies and to ensure standards are met. Additional indicators of quality may also need to be developed to reflect specific issues such as the cultural respectfulness of services*[1].

Australia’s National Mental Health Strategy has emphasised the quality, effectiveness and efficiency of services, and has promoted the collection of outcomes data as a means of monitoring these. All public sector mental health services across Australia now routinely report outcomes. Since late 2003, the Australian Mental Health Outcomes and Classification Network has received, processed, analysed and reported on outcome data at a national level, and played a training and service development role [10]. Australian governments have invested a great deal of money and effort in developing a national approach to evaluating mental health service outcomes. Despite this massive effort nothing can be said about outcomes for immigrant and refugee clients of mental health services since CALD variables are not part of the national outcomes data collection process.

### Mental health of caregivers from CALD background

Among CALD communities families are generally required to take the primary role of care giving fora relative with mental illness [121]. However, despite the demanding nature of the care giving role for CALD caregivers, there is scarce evidence on the mental wellbeing of these caregivers in Australia. A study examined the health and social experiences of Greek families with care giving responsibilities for their co-resident family member who had physical and/or mental disorder in Melbourne [122]. The majority of caregivers reported their psychological wellbeing as being worse than that of other people, and also worse than their physical health. This was attributed to the burden of care giving, which overwhelmed their ability to cope. They also reported persistent worries about their current caring role and the prospect and resources for continuing care in the future. Furthermore, caregivers of mentally ill family members revealed that they had limited knowledge about the disorders of care recipients and the type of assistance they should provide. Such lack of knowledge resulted in substantial stress and anxiety. Another study [121] explored care giving experiences for a relative with mental illness among Egyptian families living in Australia. It showed that the care giving experience in the Egyptian families was influenced by their own cultural and religious traditions. The families had a high sense of obligation and duty to provide care although they felt powerless, isolated, stigmatized, and embarrassed, and with limited support. They also had poor understanding of mental illness and had limited access to necessary information due to the language barrier. There was increased experience of depression, anxiety, and suicidal thoughts in these families.

There are very few studies of the effect of caring for people with mental illness on families from CALD background. This is a largely neglected area of research in Australia, despite the importance of understanding the perspective, beliefs and practices of immigrant and refugee carers.

### Investigator-initiated and strategic research

The research reported above is almost entirely investigator-initiated research. This is research that is conceived, designed and carried out on the initiative of individual investigators who have an interest in a particular research question, design a study that will answer the question and secure the necessary financial and other supports required to carry out the research. Such research is extremely important in all fields and is the source of innovation and scientific progress. It should continue to be encouraged and supported.

However it is clear from the above brief review that the body of research produced in this way is fragmented, partial and somewhat disconnected from the concerns of policy makers and practitioners. Investigator-initiated multicultural mental health research needs to be supplemented with strategic research that will answer questions that are important to policy makers, service designers and evaluators and practitioners.

There is a need to develop a strategic multicultural mental health research agenda. Among the questions that may be of high priority in such an agenda are the following:

•What is the prevalence of mental disorders (and of specific disorders) in the immigrant and refugee population (and in specific immigrant and refugee sub-groups)?

•What are the patterns of mental health service use in different segments of the mental health system (e.g. hospitals, community mental health, primary care, forensic, child and adolescent mental health services)?

•Which immigrant and refugee sub-groups are particularly at risk of developing mental disorders and likely to require particular attention from mental health promotion, illness prevention and mental health service programs?

•Which are the most important social determinants of mental health and illness in immigrant and refugee populations, and which of these are amenable to social policy interventions?

•Do specific immigrant and refugee populations under-use available mental health services?

•What are the determinants of patterns of mental health service use?

•What are the outcomes of contact with mental health services in meeting the needs of people with mental illness and their families and support persons, in particular for people from non-English speaking backgrounds?

A mental health research agenda has been developed for young refugees [123] by a consortium of agencies including the Centre for International Mental Health, University of Melbourne, the Victorian Foundation for Survivors of Torture, the Royal Children’s Hospital Melbourne and the Centre for Multicultural Youth. Consensus has been elicited on high priority research questions in each of nine research domains:

1. Epidemiology/prevalence of mental health problems

2. Understanding determinants of mental health (e.g. what are the key risk and protective factors)

3. Assessment of mental health problems

4. Conceptualization of “mental health/illness” and help-seeking strategies

5. Mental health service models/systems

6. Mental health services utilization

7. Treatment methods and interventions’ evaluation

8. Mental health promotion

9. Research methodology

This can be used as a basis for developing a broadly relevant multicultural mental health research agenda.

## CALD mental health data collections

*The Mental Health Service delivers services that take into account the cultural and social diversity of its consumers and meets their needs and those of their carers and community throughout all phases of care. Standard 4, National Standards for Mental Health Services 2010*[9]*.*

The implementation guidelines for Standard 4 (Diversity Responsiveness) of the National Standards for Mental Health Services 2010[9] include the following: “The MHS whenever possible utilises available and reliable data on identified diverse groups to document and regularly review the needs of its community and communicates this information to staff.” This section will examine whether national data collections support this aspect of Standard 4.

In 1999 the Australian Bureau of Statistics published the Standards for Statistics on Cultural and Language Diversity to identify, define, classify and particular attributes that relate to cultural and linguistic background [124]. The Standards were intended as a replacement for the designation ‘non-English speaking background’ (NESB), which was previously used as a broad descriptive measure.

The full set of recommended CALD variables is:

1. Indigenous status

2. Country of birth

3. Country of birth of father

4. Country of birth of mother

5. Ancestry

6. Religious affiliation

7. Year of arrival in Australia

8. Proficiency in spoken English

9. First language spoken

10. Main language spoken at home

11. Main language other than English spoken at home

12. Languages spoken at home

The minimum data set recommends four variables to capture cultural and linguistic diversity:

1. Country of birth

2. Main language other than English spoken at home

3. Proficiency in spoken English

4. Indigenous status

The Standards observe that “to use a single standard variable, such as country of birth, or a non-standard composite concept, such as NESB, is inadequate.”

We identified government and non-government agencies that collect mental health data at national or State and Territory levels and surveys that collect data relevant for mental health of immigrant and refugee populations to examine which CALD variables are included in the data collections to capture cultural diversity in Australia.

A list of agencies and surveys that collected CALD mental health data, and the variables used to capture cultural diversity, are shown in Table 2[125-128].

**Table 2:** Data Elements Relating to Cultural and Linguistic Diversity

**Agency/organization**	**Data collections/surveys**	**CALD variables**								
		**Country of birth**	**First language spoken**	**Interpreter service required**	**Main language other than English spoken at home**	**Period of residence in Australia (years)**	**Preferred language**	**Proficiency in spoken English**	**Year of first arrival in Australia**	**Country of birth of parents**
Australian bureau of statistics	Causes of death collection^b^	**√**				**√**				
	General docial survey^c^	**√**			**√**			**√**	**√**	
	Australian health survey^b^	**√**			**√**			**√**	**√**	
	National survey of mental health and Well-being(2007)^d^	**√**			**√**			**√**	**√**	**√**
	Survey of disability, ageing and carers (SDAC)^e^	**√**			**√**			**√**	**√**	
Australian Institute of family Studies ^b^	Growing up in australia: The longitudinal study of Australian children (*Ethnicity data collected for study child and all other members of the household)	**√**			**√**				**√**	
Australian Institute of health and welfare	Alcohol and other drug treatment NMDS ^a^	**√**					**√**			
	Community mental health care NMDS and Residential Mental Health Care NMDS ^a^	**√**								
	Computer assisted telephone interview demographic module DSS ^a^	**√**							**√**	
	National drug strategy household survey ^b^	**√**			**√**				**√**	
	National hospital morbidity database ^b^	**√**								
	National mortality database ^b^	**√**				**√**				
	Non-admitted patient emergency department care NMDS^a^	**√**								
	Perinatal NMDS^a^	**√**								
	National Dental Telephone Interview Survey ^b^	**√**								
Family Medicine Research Centre, University of Sydney	Bettering the Evaluation and Care of Health (BEACH)^b^	**√**								
Centre for Behavioural Research in Cancer, The Cancer Council Victoria	Australian Secondary Students Alcohol and Drug Survey^b^				**√**					
Australian Institute of Health and Welfare - General Practice Statistics and Classification Unit, University of Sydney ^b^	National Coroners Information System	**√**				**√**				
The Kirby Institute, University of New South Wales ^b^	Australian Needle and Syringe Program Survey	**√**			**√**^**c**^					
The Kirby Institute, University of New South Wales ^b^	National HIV Registry	**√**								
National Drug and Alcohol Research Centre, University of New South Wales ^b^	National Clients of Treatment Service Agencies census	**√**			**√**					
AIHW National Perinatel Epidemiology and Statistics Unit, The Perinatal and Reproductive Epidemiology Research Unit, University of New South Wales b	Perinatal Data Collection, Australia	**√**								
Women’s Health Australia^b^	The Australian Longitudinal Study on Women's Health									

^a^Souce: AIHW (2012); ^b^ Source: Blignault and Haghshenas (2005). [125]^c^ Source: ABS (2011). [126], the survey also includes 33 questions on visa category (e.g. type of visa for people who are not an Australian citizen, type of first visa, whether the person was a temporary resident before becoming an Australian citizen or permanent resident and so forth); ^d^ Source: ABS (2009) [127]^e^ Source: ABS (2010) [128].

### Gaps in CALD data collections

Current data collections by Commonwealth, State and Territory agencies and other relevant national agencies do not include most of the variables that are recommended by the Australian Bureau of Statistics Standards. This results in multiple data deficiencies.

#### Reliance on country of birth as sole indicator

As seen in Table 2 most Australian surveys and other relevant data collections, and reports based on these data collections, only make reference to ‘country of birth’. Further, country of birth is very frequently aggregated into ‘region of birth’. If language data is collected and reported, it is generally only reported as ‘English’ or ‘non-English’ [129].

In the health sector data collections the Standards are poorly implemented. A review of national surveys [125] found that:

•Seven surveys used ABS standards and classifications. Only one included all the minimum dataset variables for CALD.

•Of 17 national datasets reviewed, 12 included country of birth, three also included language but none included all three variables.

Clearly, the standards have not been implemented as intended, and ‘country of birth’ is used in isolation, without the other minimum data set variable.

The use of ‘country of birth’ as a classification of CALD populations is problematic as it is only one of several factors that may influence culture, language and ethnicity [130,131].

This is a major problem in regard to child and youth services where the identified client, i.e. the child, has “Australia” recorded as Country of Birth, while her/his overseas-born parents may not speak English. In child and youth services the family is often as involved in the receipt of services as the identified client, however the relevant cultural and language data is not captured and therefore not considered.

#### Aggregation into country of birth categories for data analysis

Many surveys, such as the Australian Health Survey, aggregate overseas born people into categories, for example by region of birth and by whether they speak ‘English’ or ‘Languages Other Than English’. Commonly used aggregate categories are listed in Table 3.

**Table 3:** Commonly used aggregate country of birth categories

**Country of**	**Year of arrival**	**Main language spoken**
**birth groups**	**groups**	**at home groups**
Australia	Arrived before 1996	English
Other Oceania	Arrived 1996-present	Language other than English
United Kingdom		
Other North-West Europe		
Southern and Eastern Europe		
North Africa and Middle East		
South-East Asia		
All other countries		

There are problems with this approach. Aggregating people into categories can average out differences and hide the most vulnerable populations [132]. For example, one British study that explored this tendency to aggregate found that the self-reported smoking prevalence for both South Asians and Europeans was 33% [133]. It appeared that there was no difference between the two groups. However, when the ‘South Asian’ category was disaggregated, it was found that the rate among Indians was 14%, among Pakistanis it was 32%, and among Bangladeshi males the rate was 57%. This illustrates how the practice of aggregating population groups can give a ‘misleading average’ [134] and obscure differences of considerable importance. The data may appear accurate but masks an important inequality [133]. A necessary targeted response is made impossible.

Aggregation can also mask differences between CALD men and women. For example, in New South Wales, the smoking prevalence among Vietnamese-born people is 16.3%. When this figure is disaggregated, it is 30% for men and 2.2% for women [135]. In Victoria, the gender impact assessment process recommends that to understand the impact of diabetes, gender sensitive or disaggregated data and reporting is required [136].

Aggregation into regions is not a useful tool for policy makers or practitioners as it does not identify which populations are at increased health risk. For example, if people born in North Africa and the Middle East are hospitalised more for a certain condition, a more in-depth mixed methods analysis is needed to identify the specific community to develop appropriate intervention strategies.

Clearly, the aggregation of data in the ways in which this is routinely done in relation to CALD populations severely limits the usefulness of the data collection and reporting. It may obscure important inequities and fail to identify important needs.

#### Insufficient CALD sample size in national surveys

One of the key reasons for aggregating country of birth groups into regional groups is the small sample sizes of the individual country of birth groupthat constitute the overall sample. The relatively small CALD sample size, even in larger surveys, limits the degree to which data can be disaggregated by subgroup. It is difficult, if not impossible, in general surveys to achieve an adequate sample size for individual country of birth groups if this is not addressed as part of the study design. A strategy that has frequently been recommended [40] to address this problem is to select CALD sub-groups that are of particular practical or theoretical interest in relation to the study and to over-sample from those groups in order to ensure that there are adequate numbers to enable meaningful, disaggregated data analysis.

#### Exclusion of people with limited or no English proficiency from national surveys

A review of Australian national data sets and surveys found that all, except for the national Census, have limited CALD sample sizes and that people with limited English proficiency are frequently explicitly excluded[125]. The additional cost associated with translation and the employment of bilingual interviewers, and the frequent unavailability of translated and validated instruments, are often given as reasons for excluding people with limited English proficiency. This exclusion is a particular concern given the association between lack of English proficiency and socioeconomic disadvantage. [125]. The frequent exclusion of a particular population group limits the generalisability of study findings. Data on regional country of birth groupings reported in national surveys are based on responses from participants who are proficient in English and who may therefore not be representative of the immigrant and refugee population of which they are part.

#### Lack of confidence concerning quality CALD data

It is difficult to find an acknowledgement in Australian research reports that there may be legitimate questions about the quality of data derived from immigrant and refugee participants. Quality issues are only highlighted in the few studies that have been conducted specifically on the issue of CALD data coverage and quality [125,137,138].

In Australia’s Health 2010 report, the description of the health of Aboriginal and Torres Strait Islander peoples includes an acknowledgement that a number of data quality issues remain unaddressed. These are described as ‘logistical, analytical and conceptual challenges’ [139]. All of these issues apply equally to immigrant and refugee participants in health studies. The Australia’s Health 2010 report does not comment on this issue in the ‘overseas-born’ section of the report.

## Representation of CALD participants in Australian mental health research

A comprehensive examination of research on depression in immigrant and refugee communities was carried out in 2002 [11]. The search for relevant research was systematic and extensive. It included a systematic search for relevant Australian studies published between 1990 and 2002, a search for higher degree theses deposited in 30 Australian University libraries, and a survey of 277 relevant university departments and research organisations to identify research under way. The search for publications yielded only 30 relevant publications, ten focusing on refugees and asylum seekers, six focusing on depression in the post-partum period, six studies of suicide rates in different immigrant and refugee communities and eight on various mental health problems in various immigrant and refugee communities. Of the 228 higher degree theses that dealt with depression only five (2.2%) addressed issues relevant to immigrant and refugee communities. Of the 277 university departments and research institutions surveyed 91 (33%) responded. Only nine relevant projects were identified. The study concludes that “the body of research published and the work currently conducted is very limited in scale and scope. Little is known about the prevalence of depression, risk factors and protective factors, cultural concepts of depression and attitudes to depression, pathways to care, and uptake and effectiveness of existing interventions in relation to CALD communities. For depression in CALD communities there is effectively no evidence base to support mental health policy development and service design, and there is virtually no evidence concerning effectiveness of services currently provided or regarding particular treatment approaches and models of service [11].”

In 2010 a systematic literature review [39] of the representation and coverage of non-English-speaking immigrants and multicultural issues in *The Medical Journal of Australia, The Australian Health Review* and *The Australian and New Zealand Journal of Public Health* found that of more than 4,000 publications over a 12 year period only 90 (2.2%) were articles primarily focused on multicultural health issues. A further 62 articles contained a major or a moderate level of consideration of multicultural issues, and 107 had a minor mention. The authors concluded that “the quantum and range of multicultural health research and evidence required for equity in policy, services, interventions and implementation is limited and uneven. Most of the original multicultural health research articles focused on newly arrived refugees, asylum seekers, Vietnamese or South East Asian communities. While there is some seminal research in respect of these represented groups, there are other communities and health issues that are essentially invisible or unrepresented in research. The limited coverage and representation of multicultural populations in research studies has implications for evidence-based health and human services policy.”

These studies by Minas et al. [11] and Garrett et al. [39] indicate that research that is relevant to CALD communities constitutes an extremely small component of Australian mental health and general health research.

In order to examine the issue of representation of CALD communities in Australian mental health research we conducted a systematic review of Australian studies published between 1992 (the commencement of the National Mental Health Strategy) and 2012 in four key Australian journals, the *Australian and New Zealand Journal of Psychiatry, Australasian Psychiatry, Australian Psychologist* and the *Medical Journal of Australia*. The purpose of the review was to explore the extent of representation of immigrant and refugee communities in Australian mental health research and to specifically explore the frequency with which people who are not proficient in English are excluded from Australian mental health research studies.

The optimisation strategy developed by Wilczynski, Haynes and Hedges [140] was used to locate mental health research published in the selected journals in searches of the following databases: Medline (Ovid), PsycINFO (Proquest) and CINAHL (EBSCO). Studies that focused on mental health and were carried out in Australia were classified into six categories:

1. Non-English speakers excluded: when the exclusion criteria clearly excluded potential subjects who were not proficient in English from the sample;

2. General mention: when the immigrant or refugee populations are mentioned descriptively but were not part of the design or analysis;

3. Cross-national study: when the study made comparisons between samples from more than one country;

4. Part of the study: when studies specifically examined immigrant or refugee samples or issues as part of the design and analysis of the study;

5. No mention immigrant or refugee communities: when immigrant or refugee populations were not mentioned in the study;

6. Indigenous: when the studies examined issues in Aboriginal/ Torres Strait Islander people.

**Figure 4:**
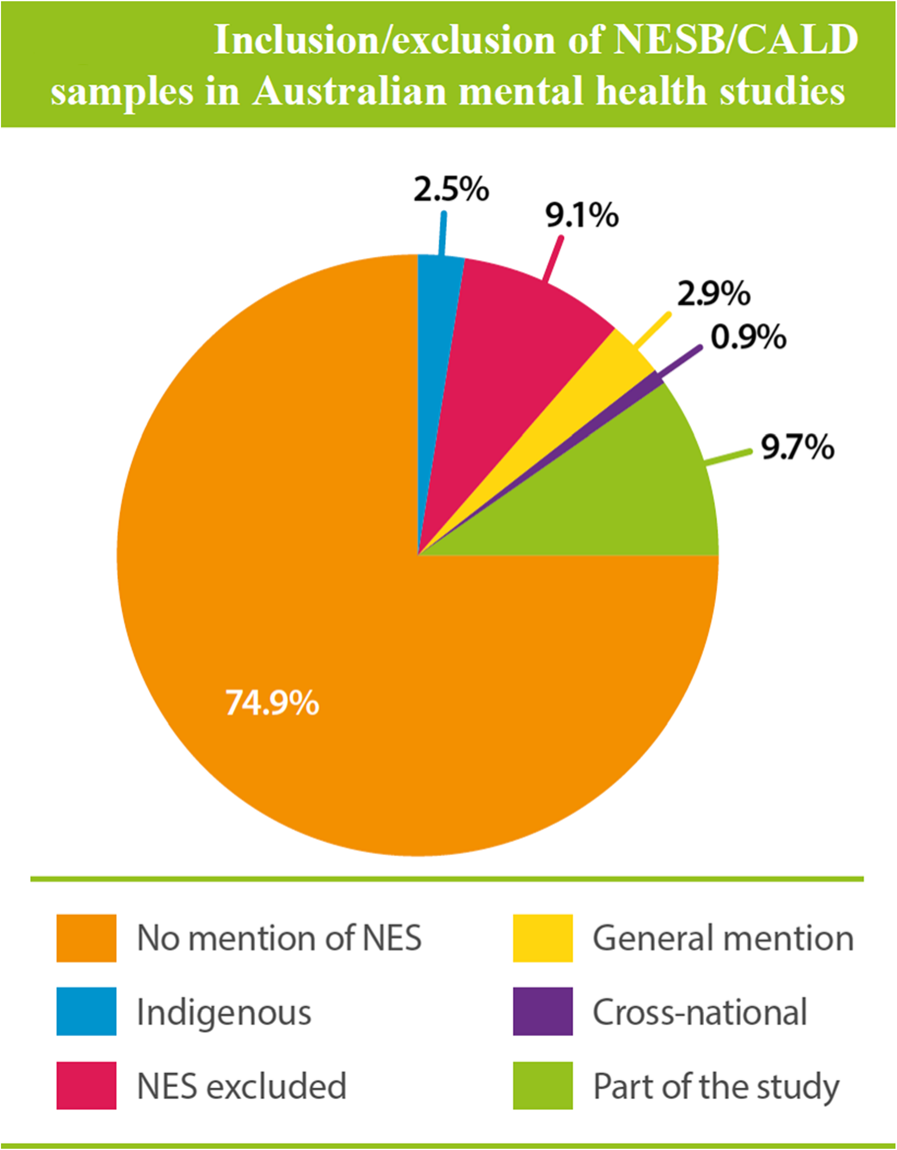
Inclusion/exclusion of NESB/CALD samples in Australian mental health studies.

Of the initial 5,545 papers identified in the search, 963 duplicates were excluded, a further 3,265 were excluded because the paper was not a report of original research, was not mental health relevant or was not an Australia-based study. A total of 1,317 papers remained and were analysed. The proportions of papers in each of the six categories are shown in Figure 4.

The great majority of papers made no mention of cultural and linguistic diversity of the populations that were being sampled and studied. In 2.9% of studies there was some general mention, but no specific analysis, of CALD issues or populations, and in 9.7% of studies there was specific consideration of CALD issues or inclusion of CALD samples. In 9.1% of studies the exclusion criteria for sampling and participation included insufficient proficiency in English.

## Research inclusion strategies in Canada, UK and USA

*Gathering evidence… requires that greater priority be given to innovative mental health research in a range of fields, including the biomedical, psychological and social sciences, program evaluation and health economics. At present, there is limited evidence as to how best to tailor assessment and treatment for specific populations, including Aboriginal and Torres Strait Islander peoples and people from culturally diverse backgrounds*[1].

In this section we briefly examine approaches taken by three countries – Canada, UK and USA – to ensure inclusion of minorities in their respective national research efforts. The historical and cultural similarities of these countries to Australia are self-evident. They are all countries with formal and long-term immigration programs, have multicultural and multilingual populations and face similar challenges in provision of effective and appropriate health and mental health services to CALD populations.

It is clear that other similar countries with multicultural populations have developed disparate strategies that aim to ensure that minority populations are included as much as possible in clinical and population mental health research. Several of the strategies, with appropriate modification, may be applicable in Australia.

### Canada

Federal agencies are required to take positive measures to ensure the support and recognition of minority language communities in Canada which, for the Canadian Institutes of Health Research (CIHR), means an obligation to promote health research that includes these communities [141]. In 2003 the House of Commons Standing Committee reported the difficulties that Official Language Minority Communities (OLMCs) experienced in accessing health services in their language of choice, and that the insufficient empirical evidence on the challenges faced by both French-speaking and English-speaking minority communities was a main barrier to the development of strategies for improving access to health services in both official languages. This led to processes designed to identify the needs for and gaps in research on the health of OLMCs and strategies for increasing the number of researchers in the field.

The outcome was a CIHR Strategic Research Initiative on OLMCs. The initiative aimed to: (1) promote the study of health determinants and specific needs of the French and English-speaking minority communities; (2) increase the number of researchers taking an interest in these issues; and (3) ensure that newly created knowledge is transmitted to researchers, clinicians and other stakeholders, with the view of improving the health of Canadian populations [142].

In Canada, a longitudinal National Population Health Survey (NPHS) household component was created to gather information about the health of general population [143] and is conducted every two years, in one of a choice of 23 languages. This survey started in 1994 (cycle 1). The most recent cycle (cycle 9) was performed in 2011. In each cycle, a common set of health questions is asked to the respondents. It includes questions about mental health and well-being, disease and health status, nutrition, health care utilisation, as well as lifestyle and socio-economic conditions. Based on the most recent questionnaire [144], respondents were able to complete the survey interview - either by telephone or face-to-face - in one of the 23 main languages spoken in Canada.

### United Kingdom

In the United Kingdom, the anti-discrimination Equality Act of 2010 serves to protect the rights of all individuals in Britain and has a clear list of “protected characteristics” such as age, disability, gender reassignment, marriage and civil partnership, pregnancy and maternity, race, religion and belief, sex and sexual orientation [145]. Although it is not an explicit directive for inclusion of minorities in research the Act does apply to a number of regulatory bodies which may engage in research activities including government departments, service providers, employers and education providers.

The National Health Service in the UK has acknowledged the importance of inclusion of “black and minority ethnic” (BME) communities [146]. It is recognised that these communities have poorer health outcomes, a shorter life expectancy and difficulty in obtaining access to health care with mental health being of particular concern [147]. A five-year action plan called the Delivering Race Equality in Mental Health (DRE) was established in 2005 with the goal of reducing inequalities for BME communities particularly in relation to their experience of and interaction with mental health services [148]. A review of the DRE identified a significant increase in the commissioning of research in these communities. According to the DRE their research has “helped us to improve ethnic monitoring, identify good practice and provide better information to patients” [148].

The Research Governance Framework for Health and Social Care [149] outlines the overarching principles of good research governance. Specifically the framework applies to research applicable to health and social care (i.e., including research relating to public health, research undertaken by the Department of Health, clinical and non-clinical research, the National Health Service (NHS) and other research bodies within the health and social care systems). According to the Framework [149]: ‘Research, and those pursuing it, should respect the diversity of human society and conditions and the multicultural nature of society. Whenever relevant, it should take account of age, disability, gender, sexual orientation, race, culture and religion in its design, undertaking, and reporting. The body of research evidence available to policy makers should reflect the diversity of the population.’

The UK has a mental health minimum dataset (MHMDS) [150] that is an approved National Health Service information standard. It was designed to deliver comprehensive, nationally consistent and comparable person-based information on people in contact with specialist secondary mental health services. It covers services provided in hospitals, outpatient clinics and in the community. The minimum dataset includes indicators of patients’ ethnicity. According to the fifth NHS Information Centre for Health and Social Care report [150] “Information on the ethnicity of people using services is now almost complete for people who spend time in hospital (97.4 per cent) and 89.5 per cent complete for people who did not spend time in hospital. This means that analysis by ethnic group is considered quite reliable”.

A national census of the ethnicity of inpatients in NHS and independent mental health and learning disability services in England and Wales has been conducted since 2005. The fifth report showed that information about ethnicity was available for 98% of all patients [151].

### United States of America

In the United States of America the National Institutes of Health (NIH) are mandated by law to ensure the inclusion of minority groups in clinical research. The inclusion of these minority groups in clinical research must be in a manner that is appropriate to the scientific question under study [152]. Minorities must be included in all clinical research studies, particularly in Phase III clinical trials, and the trials must be designed to allow valid analysis. The law explicitly states that cost is not acceptable as a reason for exclusion of minority populations. Clinical research applications that fail to include minorities without providing a valid reason may be returned without review.

Women and minorities may only be excluded if inclusion in a clinical research study is:

•inappropriate with respect to the health of the subjects;

•inappropriate with respect to the purpose of the research;

•inappropriate under such other circumstances as the Director of NIH may designate; or

•the guidelines may provide that inclusion in a trial is not required if there is substantial scientific data demonstrating that there is no significant difference between (a) the effects that the variables to be studied in the trial have on women or members of minority groups, respectively and (b) the effects that the variables have on the individuals who would serve as subjects in the trial in the event that such inclusion is not required [153].

Since 1994 NIH has continuously monitored aggregate inclusion data for study populations through a tracking system to ensure compliance with the inclusion policy. In 2002 NIH changed the format of data reporting from combined race and ethnicity format to collecting and reporting information on race and ethnicity separately [154]. This provided minimum standards for maintaining, collecting and reporting data on race and ethnicity.

## Discussion: key findings and recommendations

*The consumer has the right to have services subjected to quality assurance to identify inadequacies and to ensure standards are met*[1].

*Improvement of national data collections… will be critical to the design and refinement of services and supports, and to the identification of service gaps*[155].

There are many positive statements of policy intent in relation to immigrant and refugee communities in national mental health policies and strategies - in the Statement of Rights and Responsibilities, in the National Mental Health Standards, in multiple State and Territory policies and mental health plans, and recently in the COAG Roadmap for National Mental Health Reform.

However, there is virtually no reporting by Commonwealth or State and Territory governments concerning implementation of policy intent in relation to immigrant and refugee communities or evaluation of implementation. It is not possible to determine whether there has been any improvement in immigrant and refugee community mental health, access to mental health services or outcomes of contact with mental health services.

Investigator-initiated research on mental health of immigrant and refugee communities has yielded important insights into the mental health of particular immigrant and refugee communities, determinants of mental health and illness and patterns of use of mental health services. However this research is limited and does not provide a coherent account of the mental health of Australia’s CALD population. Nor does it answer critically important policy- and practice-relevant questions. An issue of particular importance in relation to CALD communities is the lack of systematic investigation and understanding of the perspectives and beliefs of families and carers concerning mental health and illness and mental health services, and the experience of members of CLD communities who come into contact with mental health services.

The most striking observation is the wide variation in findings across different immigrant and refugee communities. This variation represents a valuable and unrealised opportunity to systematically study population risk and protective factors for mental health and illness that would be of enormous theoretical and practical importance for the whole Australian population.

The majority of Australian mental health research does not adequately include immigrant and refugee samples. The number of studies that have specifically included adequate representative samples of immigrant and refugee populations or that have explicitly investigated multicultural mental health issues is very small. What we increasingly know about the mental health of the Australian-born population we do not know about immigrant and refugee communities.

The available evidence suggests that in, Australia, adult immigrants appear to have lower prevalence of mental illness than do the Australian-born. There is generally no difference reported in prevalence of mental disorders between immigrant and Australian-born children. It is not clear whether there is in fact a lower prevalence of mental disorders in immigrant and refugee communities or whether this conclusion is an artefact of the research methods used. Conclusions about the average prevalence of mental illness in overseas-born Australians may well be accurate, but the available data allows no conclusions to be drawn about prevalence in even the largest immigrant communities. The exclusion of immigrant and refugee participants, particularly non-English speaking persons, from national surveys and from individual epidemiological research projects does not allow any confident statement about prevalence of mental disorders in specific immigrant and refugee communities.

Factors contributing to increased risk of mental health problems in CALD populations include low proficiency in English, separate cultural identity, loss of close family bond, stresses of migration and adjustment to the new country, limited knowledge of the health system, trauma exposure before migration, and limited opportunity to appropriately use occupational skills. Factors that appear to be protective of mental health include religion, strong social support and better English proficiency. Studies that provide information about determinants have not been systematically examined to draw reliable conclusions concerning risk and protective factors for mental health and illness or about patterns of mental health service use.

Suicide rates in CALD populations generally reflect the rates in the country of birth. Suicidal behaviours in immigrants are associated with the problematic living experiences in the host country and in the country of origin. Strong family bonds, religion and traditional values were associated with lower suicide risk. The wide variations in suicide rates across immigrant and refugee communities represents a valuable and unrealised opportunity to systematically study population risk and protective factors that may find wide application in the development of more effective suicide prevention strategies for the Australian population.

Refugees and asylum seekers are at greater risk of developing mental health problems and suicidal behaviours than is the general Australian population. Prolonged detention has been found to be associated with poorer mental health in refugees and asylum seekers, particularly among children. Other factors influencing mental health of refugees and asylum seekers include experience of human rights violations, exposure to violence and threats, on-going temporary protection visas and experience of pre-migration trauma.

Generally, immigrants, refugees and asylum seekers have lower rates of mental health service utilisation than the Australian-born. The key barriers to access to mental health services in immigrants and refugees include greater stigma attached to mental illness and limited knowledge of mental health and services relative to Australian born. There is a general and persistent pattern of low rates of use by immigrant and refugee communities of specialist mental health services. Anecdotal evidence suggests that this is also the case in psychiatric disability and disability support services provided by mental health NGOs. In the absence of reliable prevalence data for CALD populations this observation is uninterpretable. It is not known whether the low utilisation rates are due to lower prevalence of mental disorders or whether system or community level barriers to mental health service access can explain them. This makes it impossible to determine whether the repeatedly stated policy intent of national, State and Territory mental health policies and plans concerning access to services and equity of service provision has been achieved.

The Council of Australian Governments (COAG) has acknowledged the general weakness of evidence for mental health system reform [155]. “There is a need to continue research and data development to improve our collective knowledge and understanding of mental health and wellbeing, the many factors contributing to it, their interaction, and effective ways to improve and maintain mental health for people across the population. For example, current Australian mental health and broader health data collections are inadequate in their description of the mental health and social and emotional wellbeing of Aboriginal and Torres Strait Islander people.” Despite the identification throughout the Roadmap of the need for specific strategies to respond to the needs of people from culturally and linguistically backgrounds there is no similar acknowledgment of the deficiencies in data concerning immigrant and refugee populations [155]. Under the section title Monitoring the Journey the Roadmap states that “*Where data is available*, (emphasis added) they will consider outcomes and progress for different parts of the community, particularly Aboriginal and Torres Strait Islander people, as well as by factors such as age group, gender, language and cultural background, socioeconomic status and location (e.g. urban or remote areas).” The key finding of this paper is that in relation to immigrant and refugee communities the necessary data are not available.

### Key findings and recommendations

The key findings are highlighted here and a recommendation is made in relation to each key finding.

#### Population diversity

Population projections are clear. Immigration, including a significantly increased humanitarian intake, will be a continuing major contributor to Australia’s future, as well as being a significant challenge to the provision of all kinds of human services, including mental health services.

##### Recommendation 1

Ensure that the increasing cultural and linguistic diversity of the Australian population is a core consideration in all mental health policy-making and funding for policy implementation of mental health service design, delivery and evaluation. This will require the full participation of representatives of immigrant and refugee communities and people with mental illness and their families and support persons in policy making and implementation processes.

#### Implementation of policies

Although attention to population diversity is a feature of most mental health policies policy statements are not translated into implementation objectives, funding is not made available to support implementation, and there is no adequate reporting of progress against policy intent in relation to immigrant and refugee communities.

##### Recommendation 2

Translate mental health policy statements that are relevant to CALD communities into explicit implementation objectives and identify funds and other resources that are needed to support implementation activities and programs that will achieve CALD mental health policy objectives, and report on progress on policy objectives in relation to immigrants and refugees.

#### Availability of prevalence data

Available research findings on prevalence of mental disorders in immigrant and refugee populations are incomplete and contradictory. There is no comprehensive Australian study of prevalence of mental disorders in immigrant and refugee populations that is adequate in scale and that enables valid disaggregation (e.g. by country of birth language or duration of residence groups) in the analysis of results. Future research that includes representative samples of at least some immigrant and refugee populations is required to address this issue.

The commonly reported observation that prevalence of mental disorders in refugee and asylum seeker communities is higher than that of the general Australian population is based on small-scale studies that often have methodological problems. Although there are many reasons to expect that prevalence in these groups will be higher larger, more comprehensive and methodologically rigorous studies are required before there can be confidence in the accuracy of the findings of higher prevalence.

##### Recommendation 3

Ensure that national surveys of mental health include representative samples of at least some non-English speaking background populations to improve population relevance of findings.

#### Determinants of mental health and illness

The evidence on determinants, explanatory models of illness, attitudes and beliefs concerning help-seeking and mental health services, is sparse, fragmented and based on small-scale studies of very few immigrant and refugee communities. A better understanding of determinants of mental health and illness in CALD populations, and of explanatory models of illness, beliefs and attitudes towards mental disorders and mental health services that includes the perspectives of family members, carers and support workers is a pre-condition for development of effective policy and effective mental health promotion and prevention, and mental health service programs.

##### Recommendation 4

Allocate high priority to research on the determinants of mental health and illness; explanatory models of mental illness; beliefs, knowledge and attitudes towards health services; and help-seeking among immigrant and refugee communities. This requires a particular focus on perspective and beliefs, and full involvement, of people with mental illness and their families and support persons in the investigation of the experience of members of CALD communities who have come into contact with mental health services.

#### Mental health service utilisation

There is quite good information on patterns of use of public specialist hospital and community mental health services. This research consistently shows that certain (particularly Asian) immigrant and refugee communities use mental health services at significantly lower rates than do the Australia-born. Although this is frequently reported as service ‘under-utilisation’ this interpretation of the observed patterns of mental health service use is not justifiable in the absence of reliable prevalence estimates and need-for-service data. Such data are required before judgments about whether utilisation rates are consistent with service needs.

Although there is a great deal of comment on probable reasons for underutilisation of mental health services by many immigrant and refugee communities there is very little research on the factors that influence patterns of services use. In particular there is little research on the influence of family and carer perspectives and beliefs, and prior experience of mental health services, on help-seeking and service access pathways.

There is virtually no data on immigrant and refugee community utilisation of mental health services provided through primary care, specialist private mental health services and psychiatric disability and rehabilitation support services.

##### Recommendation 5

Ensure adequate reporting of patterns of use of mental health services, and the experience of mental health services, of immigrant and refugee communities as part of the national mental health policy reporting framework.

#### Strategic research and evaluation

While investigator-initiated research has provided valuable information on many aspects of the mental health of immigrant and refugee communities it has not provided answers to questions that are of most importance to policy-makers, service designers, managers, evaluators and practitioners. Although investigator-initiated research is an essential component of any research enterprise, and must continue to be encouraged and supported, it should be complemented by a program of strategic policy- and practice-relevant multicultural mental health research to deal with the fact that immigrant and refugee communities are effectively excluded from the national mental health research and evaluation enterprise. The impact of this exclusion is that there are large and persisting gaps in knowledge about mental health of immigrant and refugee communities. Closing these gaps will require a systematic and targeted approach.

##### Recommendation 6

Develop a multicultural mental health research agenda that will serve as a guide to researchers, research students and research funders concerning high priority, policy- and practice-relevant research. Immigrant and refugee communities and people with mental illness and their families and support persons should be fully involved in the development of such a research agenda.

#### Minimum CALD dataset

The systematic absence of key CALD variables from virtually all Commonwealth, State and Territory funded data collections is a clear indication of the low national priority that is accorded to the mental health of Australia’s immigrant and refugee communities. This absence, or exclusion, ensures that what we increasingly know about the mental health of the general community, and the effectiveness of mental health services for the general community, we systematically do not know about immigrant and refugee communities, particular those among them who do not speak fluent English. The failure to collect CALD-relevant data as part of the national program of outcomes data collection is one of the most important and glaring gaps in CALD mental health data collections. This makes it impossible to evaluate the effectiveness of mental health services received by immigrant and refugee communities, care utilisation and continuity of care.

##### Recommendation 7

Ensure that mental health data collections include CALD-relevant variables and that these are analysed to inform our understanding of mental health in immigrant and refugee communities and the impact of mental health services and suicide prevention programs in meeting the needs of CALD populations. It is particularly important to include a comprehensive list of CALD variables in all outcome data collections, and include reporting of outcomes for immigrant and refugee clients of mental health services as part of national reporting of service outcomes.

#### Research funding

Applications to major Australian research funding organisations for funding of clinical or population mental health research can currently be made without reference to the cultural and linguistic diversity of the Australian population in the research design. Potential immigrant and refugee participants, particularly those who do not speak fluent English, can be and often are excluded from the research on the basis that inclusion is technically difficult and increases the cost of research. This perpetuates a culture of exclusion of immigrant and refugee communities from the Australian mental health research enterprise.

##### Recommendation 8

Engage major research funding organisations to develop consensus about the minimum CALD-relevant demographic variables that should be included in clinical and population mental health research studies and to develop strategies that will improve the level of inclusion of immigrant and refugee participants in Australian clinical and population mental health research.

We would suggest that implementation of these recommendations, which will require the joint efforts of many agencies and individuals will greatly contribute to the development of a culture of inclusion of all Australians in mental health research and evaluation and will enable the development of mental health policies, services and practices that will benefit all Australians.

Cultural pluralism confronts societies with a series of important challenges. These challenges include issues of distribution of resources; the legitimate role of government; and the purposes, structure and operations of social institutions, including health services.

The concept of equity in health is based on an ethical notion of fairness. Inequities in health arise when disparities in health status between two groups are considered avoidable, unacceptable, and unfair. Individuals should be able to attain their full health potential regardless of age, gender, race, or socio-economic circumstances.

Social justice and fundamental human rights lie at the heart of health equity. Inequities in health deserve our attention for both ethical and pragmatic reasons [156]. If it is the case that cultural minority groups are subject to systematic disadvantage as a result of social arrangements, including the conduct of mental health research and the organisation and delivery of mental health services, then a just society will change the social arrangements that result in such disadvantage.

The collection and analysis of health status data is central to moving toward equity in health. The disturbing absence of population-based mental health data concerning immigrant and refugee communities is in itself a great inequity in health. The dearth of mental health information about large segments of the population renders their health status and the possible deficiencies in performance of the mental health system invisible. Such problems must be brought to light to enable the development of strategies to reduce inequities in mental health status and in provision of effective mental health services.

Currently, in Australia, there exist major deficiencies in data and information on mental health status, mental health determinants, mental health service provision, and quality of service outcomes in immigrant and refugee communities. As a result it is difficult to set equity-oriented objectives and targets and to monitor and evaluate policy and service initiatives, or to estimate the personal, social and economic costs of doing nothing to rectify this situation or of interventions that will achieve policy objectives.

Although proposed actions are framed as recommendations they are not directed at specific agencies. The intent of the recommendations that have been made is to suggest strategies that will contribute to the development of a culture of inclusion of all Australians in the national mental health research enterprise. Commonwealth and State/Territory governments and many agencies and individual researchers will need to act if we are to collectively develop a culture of inclusion to ensure that Australian mental health research reflects the great cultural and linguistic diversity of the Australia population.

## Competing interests

The authors declare that they have no competing interests.

## Authors’ contributions

HM conceived, designed and directed the project, wrote the first draft and the final version. LST, RK and HM carried out the literature reviews and the assessment of levels of inclusion of CALD participants in research studies. LST, HV, SO and RP-I examined databases to identify inclusion or exclusion of CALD-relevant variables. All authors, contributed to revisions of drafts of the manuscript and to developing responses to reviews of the manuscript by the Australian Bureau of Statistics, the Australian Institute of Health and Welfare, the Commonwealth Department of Health and Ageing and the National Mental Health Commission. All authors have read and approved the manuscript.
